# Nanoparticle-Composed Photosensitive Thin Films Based on ZnO

**DOI:** 10.3390/ma17235773

**Published:** 2024-11-25

**Authors:** Tina Dilova, Anna Dikovska, Aleksandra Baeva, Genoveva Atanasova, Georgi Avdeev, Tsanislava Genova, Nikolay Nedyalkov

**Affiliations:** 1 Institute of General and Inorganic Chemistry, Bulgarian Academy of Sciences, Acad. G. Bonchev Str., bl.11, 1113 Sofia, Bulgaria; alexandravbaeva@gmail.com (A.B.); genoveva@svr.igic.bas.bg (G.A.); 2 Institute of Electronics, Bulgarian Academy of Sciences, 72 Tsarigradsko Chaussee, 1784 Sofia, Bulgaria; dikovska@ie.bas.bg (A.D.); ts.genova@gmail.com (T.G.); nned@ie.bas.bg (N.N.); 3 Rostislaw Kaischew Institute of Physical Chemistry, Bulgarian Academy of Sciences, Acad. G. Bonchev Str., bl.11, 1113 Sofia, Bulgaria; g_avdeev@ipc.bas.bg

**Keywords:** PLD in open air, nanoparticles, porous structures, composites, photosensitive properties

## Abstract

In this work, atmospheric pulsed laser deposition was used to prepare photosensitive elements. This technology is a practical and relatively inexpensive way of obtaining highly porous nanostructures composed of nanoparticles or nanoaggregates characterized by a large surface-to-volume ratio. Samples were produced via laser nanosecond or picosecond laser ablation of pure ZnO or mixed ZnO-TiO_2_ targets on quartz substrates with pre-deposited gold electrodes. The structure, morphology, optical, and electrical properties of the nanostructures obtained were studied regarding the sample composition and laser ablation regime applied. The ablation of a mixed ZnO-TiO_2_ target led to the fabrication of composite samples consisting of ZnO and Zn_2_TiO_4_ nanoparticles. The electrical properties of pure and composite samples were studied under exposure to UV light irradiation. It was found that the photosensitive properties of the samples depended on the ablation regime applied. The dark current measured for the nanosecond-deposited samples was a few nA, which was an order of magnitude larger compared to the picosecond-deposited samples. The value of the photogenerated current of the nanosecond-deposited samples was 10^3^-times higher than that of the picosecond-deposited samples. This is due to the lower absorption of the picosecond-deposited samples, as well as to the presence of defect-related radiative recombination in the picosecond-deposited samples, which limits the photocurrent rise. The estimated rise and decay times were longer for the composite samples independently of the deposition regime applied.

## 1. Introduction

Metal oxide semiconductors and, in particular, zinc oxide (ZnO) in various structures—nanoparticles, nanotubes, nanowires, and nanorods—are increasingly used in optics, biology, catalysts, etc. [1,2,3,4]. Their unique optical and electrical properties, low cost, high sensitivity, and stability make ZnO and the ZnO-based materials also promising for photodetection applications. Devices such as ZnO-based UV light photodetectors can be used in a variety of civil and military applications, environmental monitoring, biological analysis, UV astronomy, and flame sensing [5]. Over the years, various configurations of ZnO-based photodetectors have been prepared and characterized by researchers. It was found that the properties of nanostructured ZnO can be modified and improved by doping with different elements. One of the effective approaches to improve the properties of photodetectors is doping with transition elements (Cu, Co, Ni, and Ti) [6,7,8] and noble metals (Au, Ag, etc.) [9,10]. However, the noble metals are relatively expensive and, therefore, other approaches are being sought. One approach used by scientists that allows them to change the devices’ performance and meet specific application needs is to combine metal oxides rather than metals and fabricate composites. Metal oxides are significantly cheaper than noble metals and, at the same time, are high-quality materials in what concerns obtaining sensitive layers. In addition, combining two materials with different properties allows one to vary the ratios of the different oxides; thus, the properties of the composite could be adjusted. These new compounds may exhibit new characteristics that improve the detectors’ performance.

Like ZnO, titanium oxide (TiO_2_) is one of the most studied semiconductor oxide materials for applications in optoelectronic devices, as it is able to form a variety of nanostructures under different experimental conditions [11]. TiO_2_ is a transition metal oxide with a wide band gap that has several advantages, including being non-toxic, biocompatible, cost-effective, and easy to synthesize. Titanium oxide is a promising candidate for fabrication of nanocomposites that show good photosensitive properties.

The fabrication technology is of particular importance to the quality and properties of the resulting structures. ZnO and ZnO-based composite nanostructures can be synthesized by using various methods—chemical bath deposition (CBD) [12,13], spray pyrolysis, sol–gel deposition, electrochemical deposition, chemical vapor deposition (CVD), hydrothermal deposition [14], magnetron sputtering [15], and pulsed laser deposition (PLD) [16,17,18], as well as via combinations of different synthesis methods [19]. Among the deposition methods mentioned, pulsed laser deposition is one of the highly efficient approaches for the synthesis of nanostructures. In recent years, pulsed laser deposition in air at atmospheric pressure (open-air PLD or atmospheric PLD) has emerged as a promising technique for obtaining highly porous nanostructures. The material initially ejected from a target via laser ablation further evolves in the air at atmospheric pressure and forms nanoparticles of various sizes. This technology does not require a vacuum system as does the traditional PLD. Also, the technology allows easy and inexpensive preparation of high-porosity and high-purity nanostructures composed of nanoparticles/or nanoaggregates with a high surface-to-volume ratio [20,21,22]. Further, using short (nanosecond) or ultra-short (pico- or femtosecond) laser pulse durations, one can produce nanoparticles with different sizes and size distributions [20,21,22].

In this work, we focused our attention on fabricating composite nanostructures based on ZnO with the aim of finding possible applications as photosensitive elements. Samples were produced via nanosecond (*ns*) and picosecond (*ps*) laser ablation performed in air at atmospheric pressure of pure ZnO and mixed ZnO-TiO_2_ targets. We studied the structure, morphology, and the optical and electrical properties of the nanostructures obtained in what concerns the sample composition and laser ablation regime applied and found that the photosensitive properties of the samples depended on the latter. When compared to the *ps*-deposited samples, the dark current measured for the *ns*-deposited samples was an order of magnitude greater. Further, the photocurrent of the *ns*-deposited samples was 10^3^-times higher than that of the *ps*-deposited samples due to the lower absorption of the *ps*-deposited samples and the presence of defect-related radiative recombination. Regardless of the deposition regime applied, the composite samples exhibited a higher photogenerated current under UV irradiation. However, the estimated rise and decay times of the composite samples were longer.

## 2. Materials and Methods

### 2.1. Experimental

The photosensitive elements were fabricated by using a standard on-axis PLD configuration for material deposition. The experiments were carried out with *ns* or *ps* laser ablation of homemade ceramic targets at the wavelength of 1064 nm. The *ns* ablation was performed by using a Q-switched Nd:YAG laser (LS-2147, Lotis TII, Minsk, Belarus) with a pulse duration of 15 ns. The *ps* ablation process was carried out using a mode-locked picosecond Nd:YAG laser (PS-A1-1064, CNL laser, Changchun, China) with a pulse duration of 10 ps. Two types of targets were used for ablation—targets of pure ZnO and mixed targets synthesized from ZnO and TiO_2_ powders with a ratio of 5 wt% TiO_2_ in ZnO. The laser fluences applied on the target for *ns* and *ps* ablation were 10 J/cm^2^ and 0.5 J/cm^2^, respectively. The target–substrate distance was kept at 10 mm. The material ablated from the target was deposited on quartz substrates for structural and optical measurements and on quartz substrates with thermally evaporated gold electrodes for electrical measurements. The distance between the Au electrodes on the quartz substrates was 1 mm. All depositions were performed at room temperature in air at atmospheric pressure. After deposition, all samples were annealed at 300 °C in air.

### 2.2. Sample Characterization

X-ray diffraction (XRD) on an Empyrean diffractometer (PANalytical, Malvern, UK) was used to explore the samples’ crystalline structure and phase composition. The crystalline phases were identified using the PAN-ICSD and COD database cards. The lattice parameters, volume fraction, and crystallite size were obtained via Rietveld refinement of the XRD patterns by using the HighScore Plus 4.0 and ReX v.0.9.4 software [23,24]. The microstructure and crystallinity of the samples were revealed via transmission electron microscopy (TEM) using a JEOL JEM 2100 system (Akishima-Shi, Tokyo, Japan). To prepare the samples for TEM analysis, a small amount of the material was transferred to a Cu grid after the sample surface was scraped in a drop of distilled water. A LYRA I XMU (Tescan, Brno, Czech Republic) scanning electron microscope (SEM) was utilized to characterize the surface morphology of the deposited samples. The optical properties of the deposited samples were analyzed by studying their transmission and reflection spectra in the UV–near IR ranges by using a Lambda1050 spectrophotometer (PerkinElmer, Waltham, MA, USA) equipped with an integrating sphere. In addition to the optical properties, the sample’s photoluminescence was examined with a FluoroLog 3 spectrofluorometer (HORIBA Jobin Yvon, Glasgow, UK) with a 325 nm excitation light.

**Figure 1:**
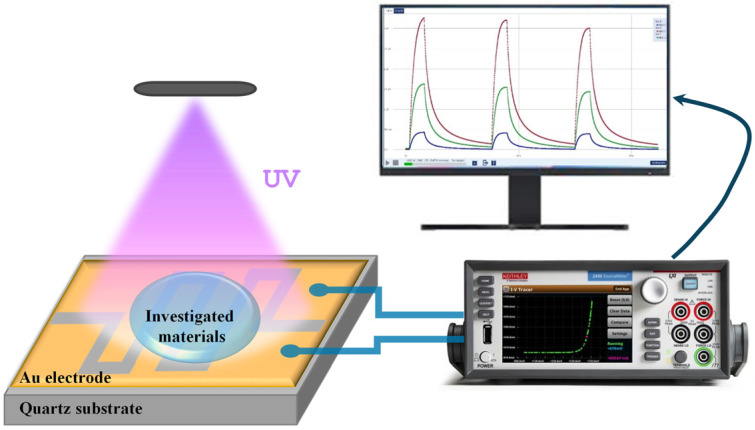
Schematic view of the photosensitive element and experimental setup for photosensitive measurements.

The electrical properties were measured via a digital multimeter Source Meter Keithley 2450 unit connected to a PC for data analysis. The photosensitivity measurements were carried out using the experimental setup shown in Figure 1 under UV-light illumination (λ = 396 nm) with different power intensities varying from 33 mW/cm^2^ to 84 mW/cm^2^. The main parameters determined via the electrical measurements of the photosensitive elements were the current gain (*G*) and the rise (*τ*_rise_) and decay (*τ*_decay_) time. The gain was determined as follows:

*G* = *I_ph_*/*I_dark_*,

where *I_ph_* is the current measured under UV light illumination, and *I_dark_* is the current measured in dark. The rise and decay time were determined as *τ*_rise_ = *τ*_90%_ − *τ*_10%_ and *τ*_decay_ = *τ*_10%_ − *τ*_90%_, where *τ*_10%_ and *τ*_90%_ are the time at 10% and 90% of the current saturation value.

## 3. Results

**Figure 2:**
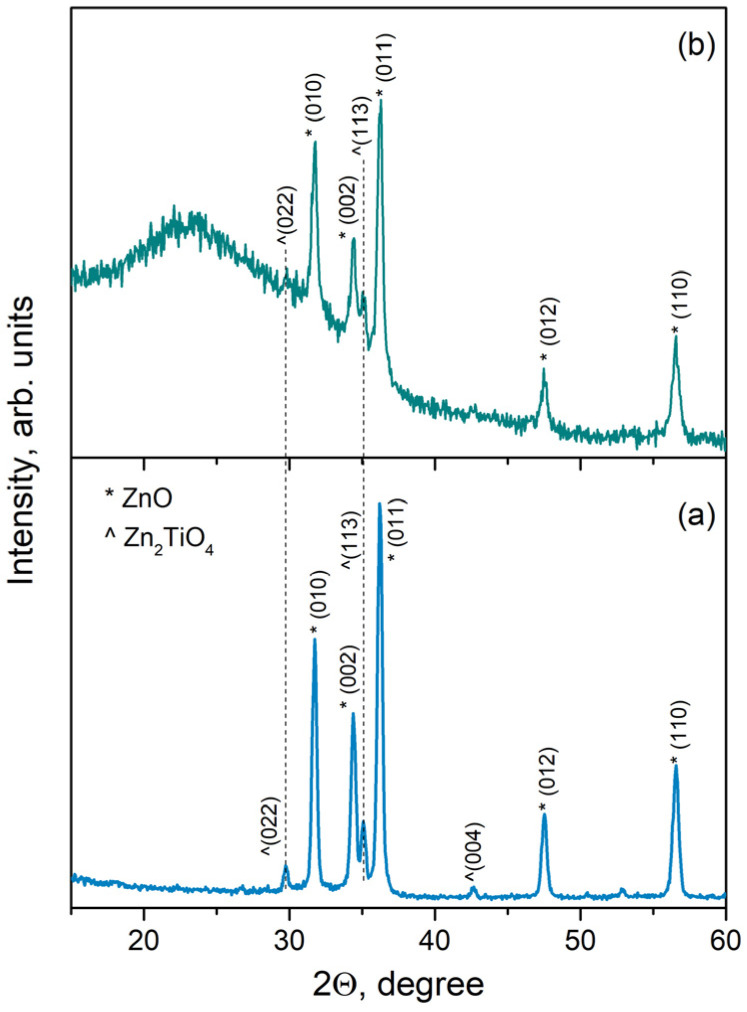
XRD patterns of the samples produced via atmospheric PLD using a mixed ZnO-TiO_2_ target with (**a**) *ns* and (**b**) *ps* laser pulses.

Our previous research has shown that atmospheric PLD of a pure ZnO target regardless of the ablation regime applied (*ns* or *ps* ablation) leads to the deposition of samples with a composition identified as a hexagonal structure of bulk ZnO (ICSD 98-007-6641, a = 3.2500 Å, and c = 5.2070 Å) [20,21]. XRD patterns of the samples deposited from mixed ZnO-TiO_2_ target are presented in Figure 2. Figure 2a shows the XRD pattern of the sample deposited via *ns* ablation. The phase composition was identified as a combination of ZnO and dizinc titanium oxide (Zn_2_TiO_4_, ICSD 98-008-0851, a = 8.4610 Å), which is in line with our previous report [20]. The XRD pattern of the sample deposited via *ps* ablation is presented in Figure 2b. As can be seen, both ZnO and Zn_2_TiO_4_ phases are present in the pattern, as is the case of *ns* ablation. One should note the presence of an amorphous halo in the XRD pattern of the sample deposited via *ps* ablation (Figure 2b), in contrast with that of the sample deposited via *ns* laser pulses.

The precise phase compositions of the samples deposited via *ns* and *ps* ablation of the mixed target are reported in Table S1. The composition of the target used for ablation is also presented in Table S1 as a reference value. No significant difference was observed in the phase composition of the target used and the samples deposited. Table 1 summarizes the lattice parameters and crystallite size of both phases in the samples’ structure. The samples deposited from the ZnO target are presented as reference samples. The reference values of the lattice parameters for the bulk ZnO and Zn_2_TiO_4_ materials are presented above in the text. No considerable difference is seen in the lattice parameters of the samples deposited via *ns* and *ps* ablation of the same target (pure ZnO or mixed ZnO-TiO_2_). Further, no significant difference is observed in the lattice parameters of ZnO in pure and composite samples. As seen in Table 1, the crystallite size of the samples deposited via *ns* ablation is slightly larger than that of the samples deposited via *ps* ablation.

The specific microstructure of the material ablated from the ZnO target in air at atmospheric pressure, as already reported in our previous research, consists of separate and aggregated nanoparticles [20,21]. The ablation regime applied (*ns* or *ps*) influences mainly the size and shape of the nanoparticles formed in the plasma plume [20,21].

**Figure 3:**
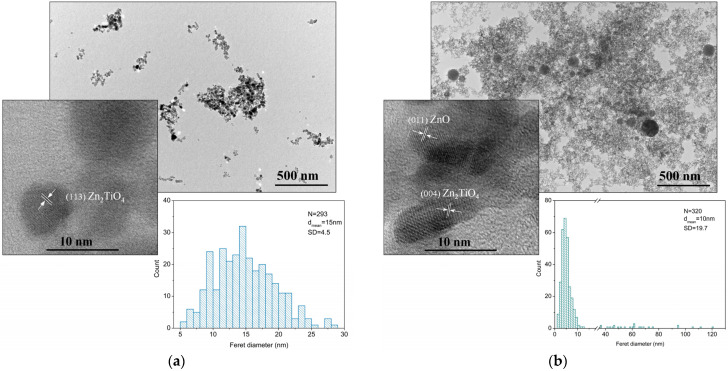
TEM image and corresponding size distribution and high-resolution TEM image of the selected area of the samples produced via atmospheric PLD using a mixed ZnO-TiO_2_ target with (**a**) *ns* and (**b**) *ps* laser pulses.

TEM images of the material ablated from a mixed ZnO-TiO_2_ target via pulses of different duration are presented in Figure 3. The microstructure of the material ablated with *ns* pulses consists of aggregated and separate nanoparticles with a shape close to spherical (Figure 3a). The size distribution also presented in Figure 3a shows that the size of the nanoparticles is in the range of 6–30 nm with a mean Feret diameter of 15 nm. The high-resolution TEM image reveals that the nanoparticles are crystalline and can be assigned to ZnO and Zn_2_TiO_4_ phases. Figure 3b shows the microstructure of the material ablated from a mixed ZnO-TiO_2_ target with *ps* laser pulses. The microstructure of the ablated material consists of spherical and irregularly shaped (elongated) nanoparticles (Figure 3b). Further, individual larger nanoparticles can be clearly distinguished with sizes ranging from 35 nm to 120 nm, together with smaller ones with sizes of 1–15 nm (Figure 3b).

The estimated mean Feret diameter of the nanoparticles is 10 nm, as the most probable diameter is approximately 5 nm. Crystalline particles identified as ZnO and Zn_2_TiO_4_ phases can be clearly distinguished in the high-resolution TEM images.

**Figure 4:**
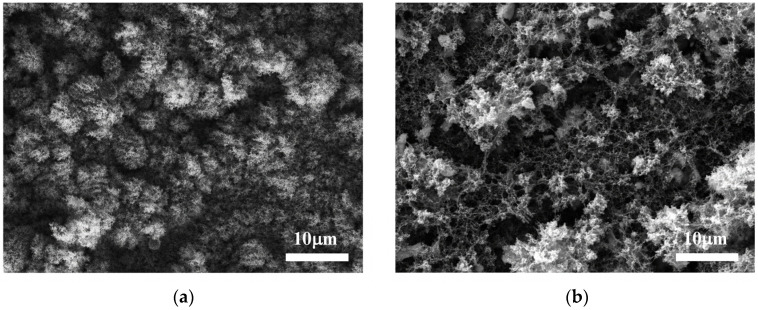
SEM image of the samples produced via atmospheric PLD using a mixed ZnO-TiO_2_ target with (**a**) *ns* and (**b**) *ps* laser pulses.

A typical morphology of the samples deposited via atmospheric PLD represents a highly porous structure formed by randomly accumulated nanoparticles with different sizes [20,21]. SEM images of samples produced with *ns* and *ps* ablation from a mixed ZnO-TiO_2_ target in air are shown in Figure 4. In both cases (*ns* and *ps* PLD), highly porous structures are formed on the substrate (Figure 4a,b). The samples’ surfaces contain meso- and macropores. Further, the samples‘ morphology does not differ substantially from that of the samples obtained via ablation of pure ZnO target independently of the ablation regime applied [20,21].

**Figure 5:**
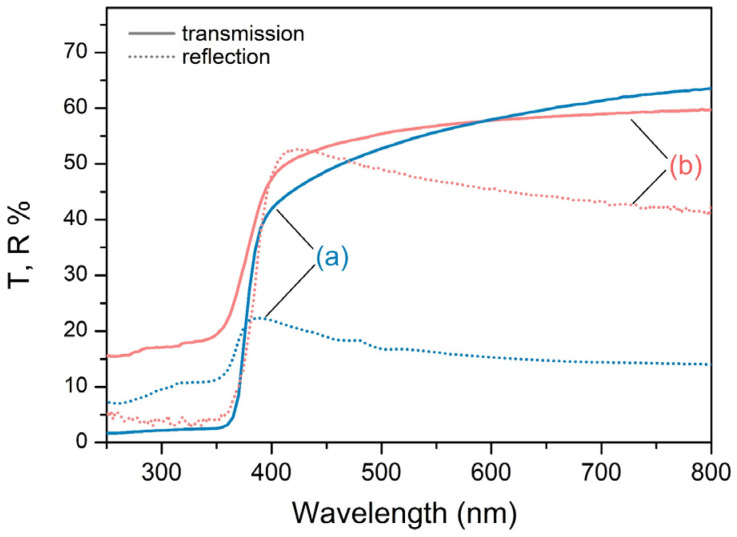
Optical properties of the samples produced via atmospheric PLD using mixed ZnO-TiO_2_ targets with (a) *ns* (blue lines) and (b) *ps* (red lines) laser pulses. The optical transmission is presented by continuous lines, and the reflection is represented by dotted lines.

Figure 5 demonstrates the optical properties of samples produced via atmospheric PLD using mixed ZnO-TiO_2_ targets and *ns* and *ps* laser pulses. The sample deposited via *ns* PLD is transparent to visible light with a sharp cut-off below 370 nm when it almost completely absorbs the incident UV light. Similarly, the sample deposited via *ps* laser pulses exhibits a visible-light transparency with a relatively sharp cut-off below 360 nm and approximately 85% UV light absorption. It is worth pointing out that the light reflection of the sample deposited via *ps* PLD is substantially higher (threefold for Vis and near IR) than the reflection of the sample deposited via *ns* PLD.

**Figure 6:**
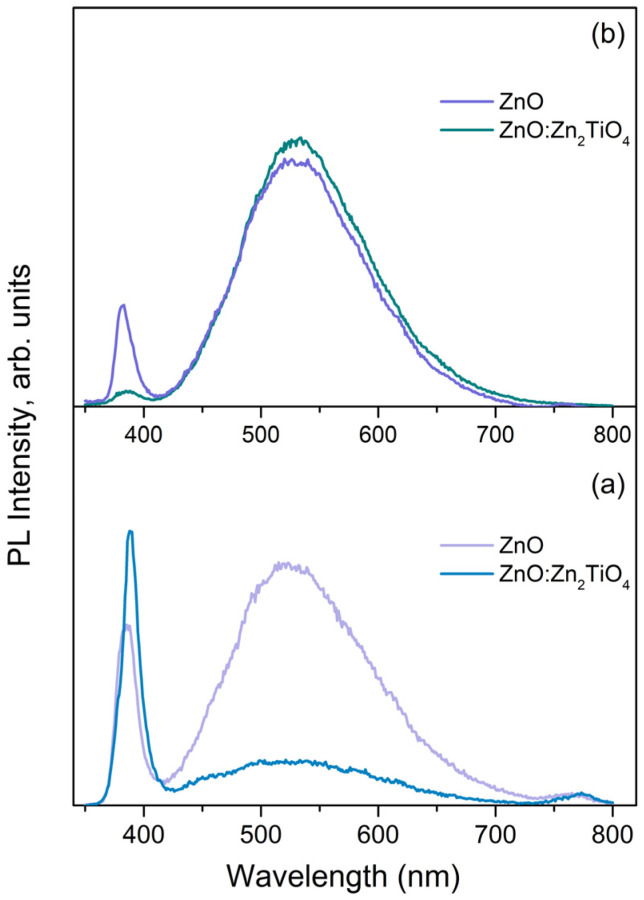
Photoluminescent properties of the samples produced via atmospheric PLD using pure ZnO and mixed ZnO-TiO_2_ targets with (**a**) *ns* and (**b**) *ps* laser pulses.

Figure 6 illustrates the photoluminescent properties of samples produced via ablation of pure ZnO and mixed ZnO-TiO_2_ targets. As seen in Figure 6a, the PL spectrum of the pure ZnO sample deposited via *ns* ablation exhibits features throughout the UV-to-IR optical range [20,21], with the visible emission being more intensive. A significant change in the sample’s PL response is observed when a small amount of Zn_2_TiO_4_ NPs is introduced to the samples’ structure (Figure 6a).

Generally, the composite sample’s PL spectrum is similar to that of the pure ZnO sample, but the ratio between the UV and VIS emission intensities is different. In the case of *ps* PLD, the ZnO sample’s PL emission (shown in Figure 6b) occurs predominantly in the visible range. It should be noted that IR emission of the *ps*-deposited ZnO sample was not observed. The composite sample (Figure 6b) deposited from the mixed target demonstrates a stronger VIS and significantly weaker emission in the UV compared to that of the pure ZnO sample. The PL response of the composite samples deposited via *ns* and *ps* ablation can be shortly summarized as follows: the samples demonstrate different photoluminescent behavior (Figure 6a,b); the composite sample fabricated via *ns* PLD emits predominantly in the UV optical range; and the composite deposited via *ps* PLD generally emits in the visible range of the spectrum.

**Figure 7:**
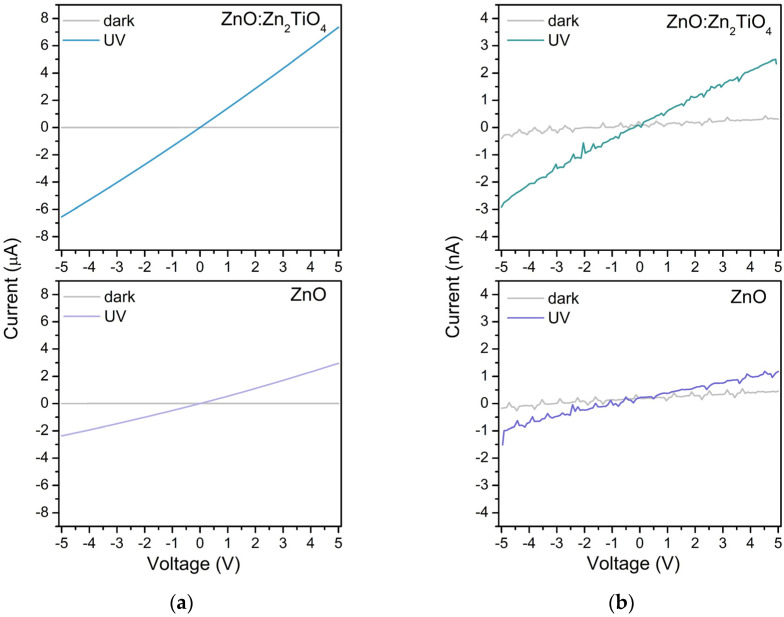
I–V characteristics in the dark and upon UV exposure of the samples produced via (**a**) *ns* and (**b**) *ps* laser pulses. The UV light intensity applied was 84 mW/cm^2^.

Figure 7 reports the current–voltage (I–V) characteristics of samples produced via ablation of pure ZnO and mixed ZnO-TiO_2_ targets. Figure 7a presents the I–V characteristics of samples deposited via *ns* laser pulses in the dark and upon UV exposure. Increasing the applied voltage leads to a rise in the device current in both cases—in the dark and under UV exposure. In addition, the photocurrent and the dark current characteristics are linear and almost symmetric at a forward and a reverse bias. In the dark, the current increase with the applied voltage is practically negligible, while the device current rises sharply when the UV is switched on. The dark current measured is about 1.43 nA and 1.28 nA at 5 V for the pure ZnO and the composite sample produced via *ns* PLD, respectively. In contrast, the photocurrent obtained for these samples under UV light illumination is about 2.96 μA and 7.35 μA, respectively, at a voltage of 5 V. The I–V characteristics of the samples deposited via *ps* laser pulses in the dark and upon UV exposure are shown in Figure 7b. As seen, the I–V behavior of these samples is similar to that of the *ns* PLD prepared ones, with the major difference being that the measured current values are orders of magnitude lower compared to the values obtained for the corresponding *ns*-deposited sample. The dark current values measured for pure ZnO and for composite samples produced via *ps* PLD are about 0.46 nA and 0.3 nA, while the photocurrent obtained under UV light illumination is about 1.18 nA and 2.5 nA at a 5 V bias, respectively. It should be pointed out that the I–V curves measured are noisy in the case of *ps*-deposited samples (as compared to the *ns* PLD deposited devices) independently of the samples’ composition and measuring regime applied.

**Figure 8:**
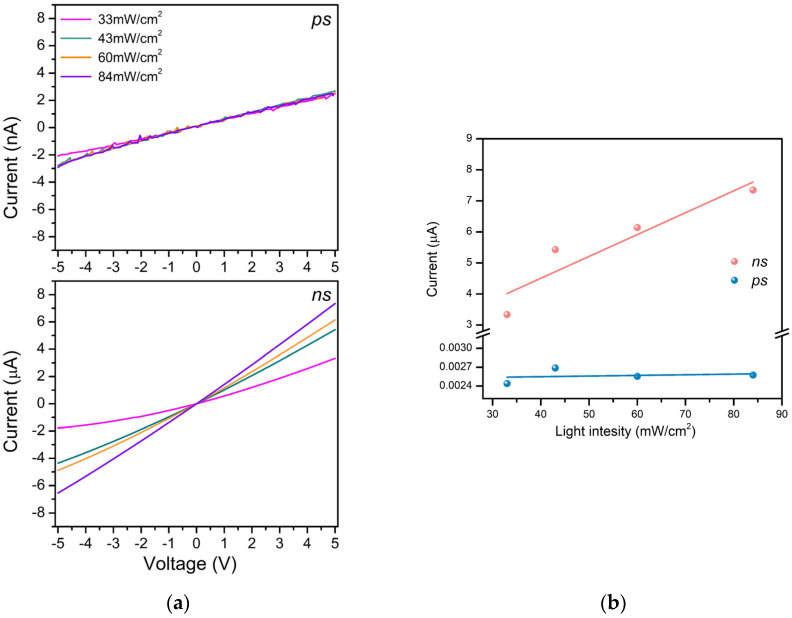
I–V characteristics of the composite ZnO:Zn_2_TiO_4_ samples produced via *ns* and *ps* ablation under different UV intensity exposures at a voltage (**a**) in the range of −5 V to 5 V and (**b**) fixed at 5 V.

Figure 8 presents the I–V characteristics of composite ZnO-TiO_2_ samples produced via *ns* and *ps* ablation upon exposure to different UV intensities. Figure 8a shows the samples’ photocurrent dependence on the UV exposure intensity as a function of the voltage in the range of −5 V to 5 V. Expectedly, increasing the illumination power density on the device surface increases the photocurrent obtained for all voltages applied in the case of *ns*-deposited samples. At the same time, the UV exposure intensity practically does not influence the value of the photocurrent for the *ps*-deposited composite sample. These results are clearly illustrated in Figure 8b, where the photocurrent behavior is shown for the *ns*- and *ps*-deposited composite samples at a fixed voltage.

**Figure 9:**
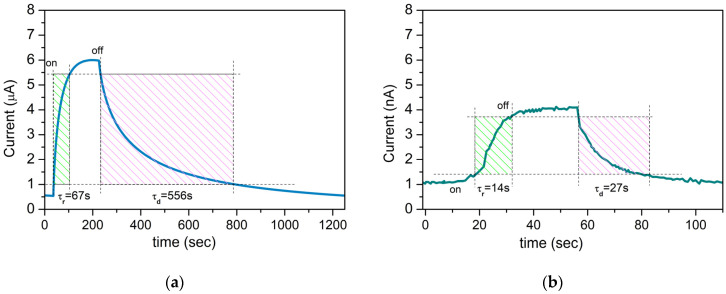
Time-dependent photocurrent response of the composite ZnO:Zn_2_TiO_4_ devices produced via (**a**) *ns* and (**b**) *ps* ablation under UV illumination with 84 mW/cm^2^ intensity at a bias voltage of 5 V. The green and pink shadow boxes show how τr and τd are defined.

The time-dependent photocurrent response of the composite devices produced via *ns* and *ps* ablation under UV illumination is presented in Figure 9a. In both cases, the photocurrent initially rises rapidly and gradually saturates under illumination.

Further, the photocurrent sharply drops when the illumination is switched off. The initial response time of the *ns*-deposited composite sample is 67 s, while the photocurrent recovers to its initial state in 556 s, approximately.

In the case of *ps* PLD, it takes about 14 s for the current to reach the maximum value when the UV light is turned on and about 27 s for it to drop to the original value when the UV light is switched off.

The photosensitivity parameters of the best samples produced in this work are summarized in Table 2; results obtained by other authors are also presented for comparison. The previously reported works selected concern ZnO or ZnO-based nanoparticulate films/structures where the photosensitivity characteristics were determined in a similar way as in this work.

## 4. Discussion

Independently of the ablation regime applied, atmospheric PLD of a mixed ZnO-TiO_2_ target leads to fabrication of a composite crystalline structure consisting of ZnO and Zn_2_TiO_4_ phases. These composite structures have a notably high content of ZnO, which is predetermined by the target used for ablation. In this work, the negligible difference in the samples’ composition (Table S1) was attributed to the error in quantification of the phase composition. It should be noted that both phases are separated and distributed as particles of different sizes and shapes, as the samples’ microstructure reveals (Figure 3).

The ZnO and Zn_2_TiO_4_ nanoparticles are clearly distinguishable in the HR-TEM images in Figure 3. However, the mean diameter of the nanoparticles produced via *ps* ablation is smaller than that of the particles fabricated with *ns* ablation (size distributions in Figure 3). In the case of *ps* ablation, a significant part of the particles produced have a size below 12 nm, while half of them have a diameter lower than 5 nm. The considerable number of such small nanoparticles (with a diameter below 5 nm) results in the appearance of an amorphous halo in the XRD pattern of the sample, since the XRD method does not recognize them as crystalline (Figure 2). Also, the presence of larger particles with sizes between 35 nm and 120 nm (size distribution in Figure 3b) points to a bi-modal nanoparticle distribution that is not clearly expressed. The latter also results in the broad nanoparticle size distribution obtained (SD = 19.7). These results are in line with our previous findings [21]. In the case of *ns* ablation, the size of the particles produced is more uniform, and the number of particles with a size below 5 nm is negligible (Figure 3a). However, the micron-sized droplets typical for *ns* PLD are also observed (not shown) [35].

The difference in the microstructure of the samples deposited via *ns* and *ps* PLD is reflected in the variation in the samples’ morphology. Thus, the morphology of the samples deposited via *ns* ablation is formed by nanoparticles with similar sizes. In contrast, a broad range of smaller-sized nanoparticles make up the structure produced via *ps* ablation. Consequently, on the one hand, the samples produced via *ps* PLD have a finer structure than their *ns*-PLD-deposited counterparts, since the former consist of smaller-sized nanoparticles. On the other hand, one could speculate that the broad range of smaller-sized nanoparticles yields a denser/smoother surface morphology since they could easily penetrate and fill the pores. This result is in line with the optical properties of the samples (Figure 5). The surface reflectance of the composite sample deposited via *ps* ablation is significantly larger than that of the sample deposited via *ns* ablation. For such small particles, light absorption prevails over light scattering.

Further, the nanoparticles formed during the ablation process in air (independently of the ablation regime applied) are collected on the substrate at room temperature at atmospheric pressure, i.e., the particles reach the substrate with low kinetic energy (due to the atmospheric pressure) possessing a low mobility on the substrate (due to the room substrate temperature). In such a manner, they are not ordered in any specific way arriving on the substrate; rather, they adhere randomly on the already deposited nanoparticles thereby forming a complex highly porous 3D structure where they are held together by van der Waals forces [36,37].

The resistance of such composite structures (consisting of touching semiconductor nanoparticles) exceeds hundreds/thousands of MΩ. Therefore, in this study, the samples were annealed in order to form grain boundary connections between the particles. Formation of interparticle sintering and grain boundary necking induced by annealing was thoroughly studied and discussed by Nasiri et al. and Liu et al. [33,36]. It should be noted that thermal annealing of such structures at the moderate temperature of 300 °C for a short time has no measurable impact on their crystalline phase structure or morphology but decreases the structure’s resistance [36]. As a result, the value measured in this work of the dark current was a few nA for the samples deposited via *ns* PLD, while for the sample produced via *ps* PLD, it was one order of magnitude smaller (Table 2). In such structures, on one hand, the numerous junction barriers are formed between particles which lower the conductance of the sample [34]. Further, the high porosity of the structures results in a small number of closely spaced particles. On the other hand, when the ZnO nanoparticles are exposed to air, oxygen molecules from the surrounding air are adsorbed on their surfaces and trap electrons from the semiconductor conduction band. This forms an electron-depleted layer on the nanoparticles’ surface, which lowers the nanoparticles’ conductance [28,33,34]. Moreover, particles of sizes smaller than twice the Debye’s length (estimated for the ZnO at room temperature to be ~19 nm) are fully depleted in the dark [29,36]. In such a manner, the smaller dark current value of the samples produced via *ps* PLD is associated with the smaller size of the nanoparticles formed during *ps* ablation and their full depletion.

When the incident UV photons are absorbed, a rise in the photocurrent is observed due to the increase in the free carrier concentration and the reduction in the depletion layer. Under UV light illumination, electron–hole pairs are formed as the photogenerated electrons accumulate in the conduction band, and photogenerated holes migrate to the surface reducing the adsorbed oxygen ions through surface electron–hole recombination [23]. As a whole, this reduces the depletion layer and increases the free-carrier concentration, resulting in the observed photocurrent rise. In other words, the conductivity is mainly controlled by the adsorption and desorption of oxygen molecules [34,38]. The high porosity of these films is beneficial in ensuring the O_2_ penetration, i.e., the formation of electron-depleted ZnO particles in the whole structure [28]. However, the photogenerated current value of *ns*-deposited samples is substantially higher than that of the *ps*-deposited samples (Figure 7a,b). This result is in line with the difference in the absorption of UV photons by the samples (Figure 5). The lower absorption of the *ps*-deposited samples produces a smaller number of photogenerated electron–hole pairs, which results in a lower free-carrier concentration (Figure 7 and Figure S1). Furthermore, the *ps*-deposited samples possess more surface defects compared to the *ns*-deposited samples, for which we assume, bearing in mind the samples’ PL behavior, particularly, the presence of intensive emission in the Vis range (Figure 6). Thus, the defect-related radiative recombination will limit a photocurrent rise under UV light illumination [39]. Therefore, it is not surprising that the increase in the intensity of the UV illumination on the surface of *ps*-PLD samples practically does not increase the photocurrent (Figure 8 and Figure S1).

Further, the presence of a small amount of Zn_2_TiO_4_ nanoparticles in the ZnO structure independently of the deposition regime applied (*ns* or *ps* PLD) increases the photocurrent (Figure 7). The formation of a composite structure based on ZnO and Zn_2_TiO_4_ adjusts the positions of the corresponding Fermi energy levels, which leads to an improvement in the effective spatial charge separation. We associate the enhanced photocurrent value of the composite samples with the formation of n-n heterojunctions [40].

When the UV light is turned off, the oxygen molecules in the air are adsorbed again onto the surface of the nanoparticles, and the UV photocurrent decays. The estimated current gain, or the on/off photo-to-dark-current ratio, of the samples deposited via *ns* PLD reaches a value 10^3^-times higher than that of the *ps*-PLD sample. Also, the on/off *I*_ph_/*I*_d_ ratio for the composite samples is higher than that for the pure ZnO samples regardless of the deposition regime applied (Figure 9 and Figure S2). However, as reported in the open literature, such porous hierarchical structures composed of nanoparticles demonstrate current gain in the range of 10^5^–10^6^ [28,29,36]. It should be noted that our samples were irradiated by light with a wavelength of 396 nm, which is practically at the edge of the visible range. Our results are comparable with the results previously reported for similar porous ZnO structures when the irradiation of the samples was carried out with a wavelength of 400 nm [28,36]. Table 2 summarizes the main characteristics of ZnO and ZnO-based composite films/structures consisting of nanoparticles obtained under UV irradiation similar to those used by us. To the best of our knowledge, the composite ZnO:Zn_2_TiO_4_ porous structure composed of nanoparticles deposited via atmospheric *ns* PLD demonstrates the highest *I*_ph_/*I*_d_ ratio under 396 nm irradiation (Table 2).

Other important parameters concerning a photodetector’s performance are the rise and decay times during the on/off cycles. The *ns*-deposited samples demonstrate substantially longer times to reach the maximum photocurrent and dark current (Figure 9 and Figure S2). Such relatively long rise and decay times are attributed to the chemi/physisorption and photodesorption of oxygen molecules on the surface of the nanoparticles [32,38,41]. Further, the rise and decay time values of pure ZnO are shorter compared to those of the composite samples for both deposition regimes applied (Figure 9 and Figure S2). This fact is probably related to the presence of more surface defects in the composite samples [22,34].

The use of such composite porous structures consisting of ZnO and Zn_2_TiO_4_ nanoparticles in view of developing photosensitive elements has not been reported. Nasiri and coauthors demonstrated the potential of tunable hierarchical photodetector structures composed of TiO_2_/SiO_2_/ZnO nanoparticles with a significant photoresponse under low applied voltage and low UV irradiation [27]. Also, the fabrication and photodetection properties of aligned ZnO-TiO_2_ and TiO_2_-ZnO core-shell nanotubes were presented by Zhou et al. [42]. The authors achieved effective separation of the photogenerated electron–hole pairs, which significantly enhanced the photocurrent. A reduced electron–hole recombination and enhanced photosensitivity characteristics were reported for hybrid structures, such as ZnO or Al-doped ZnO nanowires coated with TiO_2_ nanoparticles [41,43]. The potential of nanocomposites consisting of ZnO and Zn_2_TiO_4_ phases was previously demonstrated concerning applications as sensor elements and photocatalysts [21,44]. In the present work, the fabrication of porous composite structures consisting of ZnO and Zn_2_TiO_4_ nanoparticles in view of photodetector applications was not optimized in terms of the best device performance.

Previously, nanoparticle film/structures for photodetector application have usually been produced via a solution process or spray pyrolysis—easy and low-cost technologies based on the use of different chemical precursors and components [27,34]. Here, we delineated the potential of a technology for producing similar nanoparticulate structures by means of a physical method, i.e., without chemicals and further chemical recycling. We believe that, based on the potential of structural engineering of the nanoparticles’ morphology and composition, an improvement in the photo-to-dark-current ratio could be obtained.

## 5. Conclusions

Preliminary results are reported on the photosensitive properties of composites consisting of ZnO and Zn_2_TiO_4_ nanoparticles prepared via laser ablation of mixed ZnO-TiO_2_ targets in air at atmospheric environment and arranged in three-dimensional porous structures. We focused on the influence of the laser pulse duration used for the fabrication of photodetectors on their properties. The measured value of the dark current for the samples deposited via *ps* PLD is below 1 nA, which is smaller than the value for the *ns*-deposited sample. The smaller current value in dark for the sample produced via *ps* PLD is associated with the smaller size of the nanoparticles formed during *ps* ablation and their full depletion in air. The value of the photogenerated current of *ns*-deposited samples is 7.35 μA, which is substantially higher than that of the *ps*-deposited samples. The lower absorption and defect-related radiative recombination limit the photocurrent rise of the *ps*-deposited samples under UV light illumination. The on/off photo-to-dark-current ratio for the samples deposited via *ns* PLD is 10^3^-times higher than that of the *ps*-PLD sample. Thus, using classical *ns*-depositions resulted in obtaining a better photodetector performance. In this work, we attempt to reveal the potential of a technology for producing nanoparticle structures without the need for chemicals and chemical recycling. A better photodetector performance can be obtained using the potential of structural engineering of the nanoparticles in terms of morphology and composition.

## Figures and Tables

**Table 1:** Lattice parameters and crystallite size of the samples deposited via *ns* and *ps* ablation.

Target Used	Ablation Regime Applied		Lattice Parameter, Å	Crystallite Size, nm
ZnO	*ns* ablation		a = 3.2489	34
			c = 5.2052	
	*ps* ablation		a = 3.2473	24
			c = 5.2054	
ZnO-TiO_2_	*ns* ablation	ZnO	a = 3.2495	32
			c = 5.2050	
		Zn_2_TiO_4_	a = 8.4735	52
	*ps* ablation	ZnO	a = 3.2491	25
			c = 5.2042	
		Zn_2_TiO_4_	a = 8.4724	32

**Table 2:** Comparison of state-of-the-art UV photodetectors based on ZnO nanoparticles (NPs).

Sample	Voltage, V	λ, nm	I_d_	I_ph_	I_ph_/I_d_	Rise Time, s	Decay Time, s	Ref
ZnO NPs	5	396	1.43 nA	2.96 μA	2.07 × 10^3^	66	263	In this work
ZnO:Zn_2_TiO_4_ NPs	5	396	1.28 nA	7.35 μA	5.74 × 10^3^	67	556	In this work
5% Ni + 5% Cu:ZnO NPs	30	395	9.60 mA	10.0 mA	1.04	-	-	[8]
10% Cu:ZnO NPs	30	395	0.04 mA	0.12 mA	3.42	-	-	[8]
ZnO nanocrystals	5	395	111 μA	196 μA	1.76	-	-	[25]
ZnO nanoparticles	5	375	1.98 × 10^−8^ A	9.42 × 10^−7^ A	48	204	486	[26]
TiO_2_/SiO_2_/ZnO NPs network	1	370	22 pA	182 μA	8.2 × 10^6^	-	-	[27]
ZnO Ultraporous Nanoparticle Networks	5	370	3.61 nA	1.2 mA	3.4 × 10^5^	250	150	[28]
3D networks NPs NiO/ZnO	1	370	18 pA	344 μA	1.9 × 10^7^	5	9	[29]
ZnO colloidal NPs	120	370	2.0 × 10^−1^ nA	5.5 × 10^3^ nA	2.75 × 10^4^	-	-	[30]
ZnO nanoparticles	3	365	-	-	66.3	1.8	47.2	[31]
ZnO NPs coated with PVA	−20	340	100 pA	-	4.5 × 10^4^	-	-	[32]
Interlinked ZnO-NP networks	6	340	0.15 nA	43.6 µA	3.1 × 10^5^	9.4	13.5	[33]
ZnO nanoparticles	1	325	0.4 × 10^−12^ A	0.3 × 10^−6^ A	10^6^	48	0.9	[34]

## Data Availability

The original contributions presented in the study are included in the article/Supplementary Materials, further inquiries can be directed to the corresponding author.

## Supplementary Materials

The following supporting information can be downloaded at https://www.mdpi.com/article/10.3390/ma17235773/s1: Table S1: Composition of mixed target and corresponding samples deposited by *ns* and *ps*-ablation from it; Figure S1: I–V characteristics of ZnO samples produced via (a) *ns* and (b) *ps* ablation upon different UV exposures; Figure S2: Time-dependent photocurrent of ZnO sample produced via (a) *ns* and (b) *ps* ablation under UV illumination with 84 mW/cm^2^ intensity at 5V.
