# Supplementary material for: Nanoparticle-Composed Photosensitive Thin Films Based on ZnO
Source: Materials (Basel). 2024 Nov 25;17(23):5773. doi: 10.3390/ma17235773 (PMC11642571; doi:10.3390/ma17235773)
Supplement: Supplementary file 1 [file materials-17-05773-s001.zip › materials-3299873-supplementary.pdf]

materials

Supplementary Materials:

Article

Nanoparticle-Composed Photosensitive Thin Films Based On ZnO

Tina Dilova [1]*, Anna Dikovska [2], Aleksandra Baeva [1], Genoveva Atanasova [1], Georgi Avdeev [3], Tsanislava Genova[2] and Nikolay Nedyalkov [2]

[1] Institute of General and Inorganic Chemistry, Bulgarian Academy of Sciences, Acad. G. Bonchev str., bl. 11, 1113 Sofia, Bulgaria.; genoveva@svr.igic.bas.bg (Genoveva Atanasova); tina@svr.igic.bas.bg (T.D.); alexandravbaeva@gmail.com (A.B.)
[2] Institute of Electronics, Bulgarian Academy of Sciences, 72 Tsarigradsko Chaussee, 1784 Sofia, Bulgaria; dikovska@ie.bas.bg (A.D.); ts.genova@gmail.com (Ts.G.); nned@ie.bas.bg (N.N.)
[3] Rostislaw Kaischew Institute of Physical Chemistry, Bulgarian Academy of Sciences, Acad. G. Bonchev str., bl. 11, 1113 Sofia, Bulgaria; g_avdeev@ipc.bas.bg (Georgi Avdeev)
* Correspondence: tina@svr.igic.bas.bg

Table S1. Composition of mixed target and corresponding samples deposited by *ns* and *ps*-ablation from it.

Composition, wt%	Target	Sample deposited by *ns*-ablation	Sample deposited by *ps*-ablation
ZnO	89.3	91.7	88.2
Zn_2TiO_4	10.7	9.3	11.8

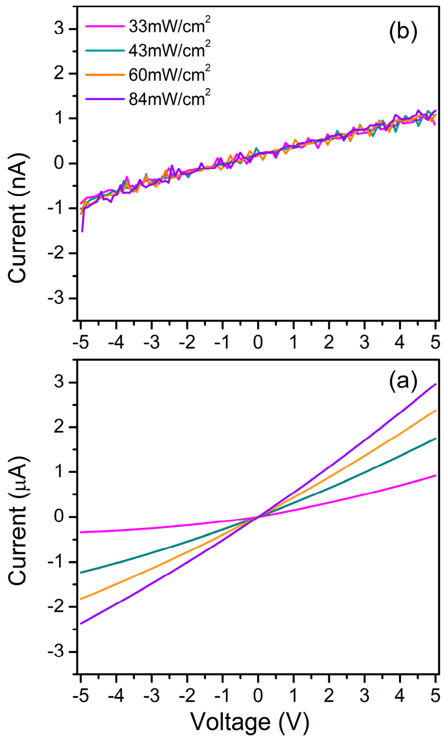

Figure S1. I-V characteristics of ZnO samples produced by (a) *ns*- and (b) *ps*-ablation upon different UV exposure.

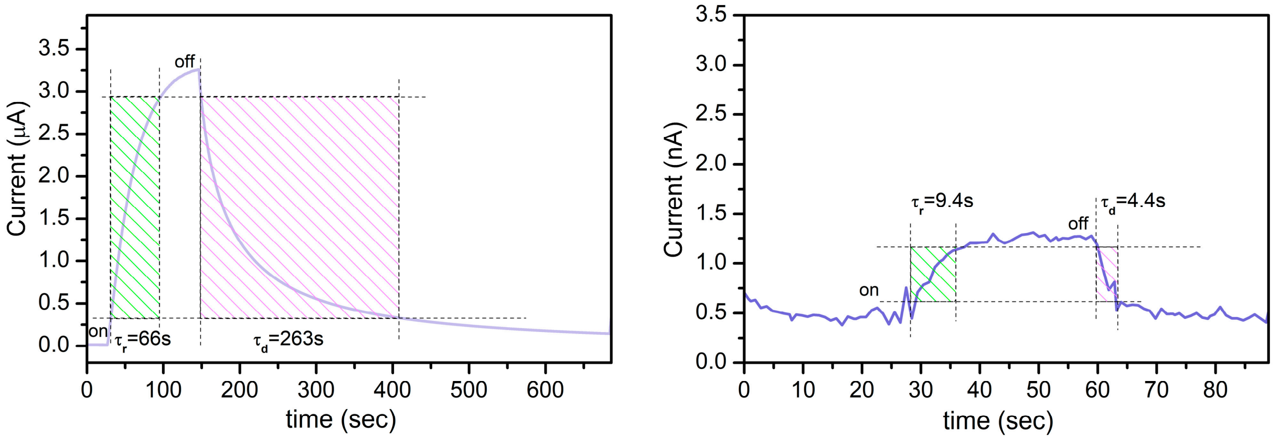

(**a**) (**b**)

Figure S2. Time-dependent photocurrent of ZnO sample produced by (a) *ns*- and (b) *ps*-ablation under UV illumination with 84 mW/cm² intensity at 5V. The green and pink shadow boxes show how τ_r and τ_d are defined.
